# ITK inhibition induced in vitro and in vivo anti-tumor activity through downregulating TCR signaling pathway in malignant T cell lymphoma

**DOI:** 10.1186/s12935-019-0754-9

**Published:** 2019-02-14

**Authors:** Yalu Liu, Xiaogan Wang, Lijuan Deng, Lingyan Ping, Yunfei Shi, Wen Zheng, Ningjing Lin, Xiaopei Wang, Meifeng Tu, Yan Xie, Weiping Liu, Zhitao Ying, Chen Zhang, Zhengying Pan, Xi Wang, Ning Ding, Yuqin Song, Jun Zhu

**Affiliations:** 1 Key Laboratory of Carcinogenesis and Translational Research (Ministry of Education), Department of Lymphoma, Peking University Cancer Hospital & Institute, 52 Fucheng Road, Haidian District, Beijing, 100142 People’s Republic of China; 2 Key Laboratory of Carcinogenesis and Translational Research (Ministry of Education), Department of Pathology, Peking University Cancer Hospital & Institute, 52 Fucheng Road, Haidian District, Beijing, 100142 People’s Republic of China; 3 Key Laboratory of Chemical Genomics, School of Chemical Biology and Biotechnology, Peking University Shenzhen Graduate School, Lishui Road, Xili, Nanshan District, Shenzhen, 518055 People’s Republic of China; 4 Department of Immunology, School of Basic Medical Sciences, Capital Medical University, 10 Xisitoutiao Road, Fengtai District, Beijing, 100069 People’s Republic of China

**Keywords:** TCR signaling, ITK, Therapeutic target, PTCL, AITL

## Abstract

**Background:**

Angioimmunoblastic T cell lymphoma (AITL) is a distinct subtype of peripheral T cell lymphoma and associated with poor outcomes. The activation status of T cell receptor (TCR) signaling has recently become a focus of attention in terms of the therapeutic targets. However, the molecular pathogenesis mechanisms and novel therapeutic targets are largely unknown.

**Methods:**

Antibodies specific to phosphorylated ZAP70, ITK and PLCγ1 were used to identify the activation status of intracellular proteins involved in TCR signaling in AITL patients. Malignant T cell lymphoma cells were transduced with a lentiviral construct containing ITK shRNA for cellular and functional assays. The antitumor effects of the selective ITK inhibitor BMS-509744 were determined in vitro and in vivo.

**Results:**

Immunohistochemistry staining showed that more than half of the AITL patients (n = 38) exhibited continuously activated intracellular TCR signaling pathway. Patients positive for phosphorylated ITK showed a lower rate of complete response (20% vs. 75%, *P *= 0.004) and a shorter progression-free survival (5.17 months vs. 25.1 months, *P *= 0.022) than patients negative for phosphorylated ITK. Genetic and pharmacological cellular ITK inhibition significantly compromised the proliferation, invasion and migration of malignant T cells. The selective ITK inhibitor BMS-509744 also induced the pro-apoptotic effects and G2/M phase cell cycle arrest in vitro and in vivo. Finally, inhibition of ITK synergistically enhanced the antitumor effect of vincristine and doxorubicin on malignant T cell lymphoma cell lines.

**Conclusions:**

Our findings suggest that ITK may be a novel candidate therapeutic target for the treatment of patients with ITK-expressing malignant T-cell lymphomas.

**Electronic supplementary material:**

The online version of this article (10.1186/s12935-019-0754-9) contains supplementary material, which is available to authorized users.

## Background

Angioimmunoblastic T cell lymphoma (AITL) is a distinct subtype that accounts for approximately 15% to 20% of peripheral T cell lymphomas (PTCLs) [1, 2]. The incidence of PTCL in Asia is substantially higher than that observed in Western countries [3, 4]. Patients with PTCL are frequently diagnosed at an advanced stage (III–IV), resulting in poor outcomes and 5-year overall survival (OS) rates ranging from 25% to 30% [1, 5]. Although the addition of etoposide to the cyclophosphamide, doxorubicin, vincristine and prednisone (CHOP) regimen and front-line stem-cell transplantation (SCT) have improved progression-free survival (PFS) in PTCL, a majority of patients, especially elderly patients, become refractory or resistant to initial chemotherapy and do not achieve long-term, disease-free survival [6, 7]. AITL derived from the malignant transformation of follicular T-helper cells is a common subtype of PTCL with unique clinical and pathological characteristics. AITL predominantly occurs in elderly people with medium age at diagnosis of 60 years [8]. Recently, Next-Generation Sequencing detected highly recurrent somatic mutations including GTPase RHOA and epigenetic regulators, which pinpointing novel candidates for the investigation of targeted therapies in AITL.

The T cell receptor (TCR) signaling pathway has gained significant attention in recent years due to its essential role in T cell development and function. TCR-dependent signal transduction is driven by activation of the Src kinase LCK, leading to phosphorylation of the CD3 immunoreceptor tyrosine-based activation motif (ITAM)-containing proteins, ZAP-70 and ITK. These kinases, in turn, phosphorylate their targets, SLP-76, LAT, and phospholipase C-γ1 (PLCγ1). Phosphorylated PLCγ1 then hydrolyzes phosphatidylinositol-4,5-bisphosphate (PIP2) to produce diacylglycerol (DAG) and inositol triphosphate (IP3), leading to increased Ca^2+^ flux and activation of NFAT transcription. The activation of the TCR also regulates several downstream signaling pathways, including the PI3K, NF-κB, MAPK, and GTPase-dependent pathways [9, 10].

Recently, several lines of evidence have suggested that the activation of the TCR pathway might also play a key role in PTCL pathogenesis. The recurrent G17V mutation in *RHOA* induces the development of T cell neoplasms by activating TCR signaling through the phosphorylation of VAV1 in AITL [11]. Furthermore, the expression of an ITK-SYK fusion tyrosine kinase was identified as a recurrent event in PTCL; this fusion tyrosine kinase acts as a powerful oncogenic driver by triggering antigen-independent phosphorylation of TCR-proximal proteins [12]. Therefore, the activation status of TCR signaling in lymphoma cells has recently become a focus of attention in terms of the therapeutic targets.

ITK is a member of Tec family (BTK, ITK, Tec, BMX and RLK), which expressed in normal T-lymphocytes and T-cell associated hematopoietic malignancies and have confirmed its critical role in regulating T lymphocyte function in EBV-driven lymphoproliferative disease and immune-mediated disorders [13–16]. Tec kinase family members shares similarities structure, consisting of PH domain, SH3 domain, SH2 domain and kinase domain [17]. Bruton tyrosine kinase (BTK) has been widely studied in B-cell hematopoietic malignancies for its critical role in B-cell receptor signaling pathway. Pharmacological inhibition of BCR signaling using the irreversible BTK inhibitor, have demonstrated notable therapeutic effects in B-cell malignancies, which shifting from chemotherapy to novel agents targeting key regulating enzymes. Thus, similar to the importance of targeting BCR signaling in B-cell malignancies, characterization of the TCR signaling status and investigation of ITK may pinpoint novel candidates for the targeted therapies in T-cell hematopoietic malignancies.

The aim of this present study was to assess the activation of TCR signaling and exploit the possible therapeutic targets or regimens for the treatment of AITL patients. Our present study illustrated that more than half of AITL patients exhibited high levels of phosphorylation of key tyrosine kinases in the TCR signaling pathway. Genetic and pharmacological inhibition of the expression of the key TCR signaling regulatory kinase ITK significantly compromised the proliferation, adhesion, invasion and migration of malignant T cells, which suggested a novel candidate therapeutic target for the treatment of AITL.

## Materials and methods

### Patients characteristics and tumor samples

A total of 38 AITL patients who were diagnosed in our Department of Pathology based on the 2016 World Health Organization classification between January 2008 and December 2017 were enrolled in this study [18]. All the patients received CHOP or CHOP-like therapy (e.g., COP, CCOP or CHOPE) as the first-line therapy. The response to the first-line therapy was evaluated after the completion of 2 to 3 courses of treatment and 1 to 2 months after the completion of all therapy, then every 3 months for the first year and every 6 months thereafter until progression. OS was calculated from the date of disease confirmation to the date of last follow-up or tumor-related death. PFS was identified as the date of disease confirmation to the date of progression or tumor-related death. The treatment response was evaluated as CR, partial response (PR), stable disease (SD) or progressive disease (PD) according to the response criteria for malignant lymphoma [19]. The clinical research protocol was approved by the Institutional Review Board and the Ethical Committee of Peking University Cancer Hospital and Institute, which were in accordance with the ethical standards of our Institutional Review Board (IRB) and with the 1975 Helsinki declaration (Revised 2010) and its later amendments or comparable ethical standards. All patients participating in this study signed the informed consent form.

### Immunohistochemistry (IHC)

Formalin-fixed, paraffin-embedded tissue specimens from 38 cases of AITL were prepared as 4-μm-thick slides by the Pathology Department for IHC. Antibodies against phospho-ZAP70 (Tyr 493, 1:100, LifeSpan BioSciences, Seattle, WA, USA), phospho-ITK (Tyr512, 1:50, LifeSpan BioSciences, Seattle, WA, USA), and phospho-PLCγ1 (Tyr783, 1:500, Cell Signaling Technology, Danvers, CA, USA) were used for immunohistochemical analysis. The immunohistochemical assay was performed by the BenchMark XT automated slide processing system (Roche, Mannheim, Germany). Each sample was graded independently by two pathologists from the department of pathology in Peking University Cancer Hospital & Institute.

The expression of the phosphoprotein was semi quantitatively estimated as the immunostaining scores. The percentage of density displayed the fraction of positive staining tumor cells (0–100%). The intensity score represented the staining intensity (‘0’ as negative, ‘1’ as weak positive, ‘2’ as moderate positive and ‘3’ as the strong positive). Slides were visualized through an Olympus BX51 microscope (Olympus, Melville, NY, USA) and photographed with Leica DM6000B camera. The images were analyzed with Leica Aperio Versa 200 (Leica Camera AG, Somme, Germany). Tumors were considered positive for phosphoprotein when ≥ 10% of the neoplastic cells demonstrated phosphoprotein staining.

### Whole exome sequencing (WES)

Sequencing libraries were generated using Agilent SureSelect Human All Exon kit (Agilent Technologies, CA, USA) following manufacturer’s recommendations and index codes were added to each sample. 150-bp paired-end reads were generated using an Illumina HiSeq platform. Reads were mapped to the reference human genome (UCSC hg19) by Burrows-Wheeler Aligner (BWA) software [20]. The somatic SNV was detected by muTect [21], the somatic InDel by Strelka [22]. Control-FREEC was used to detect somatic CNV [23]. Variants were independently identified for each tumor germline pair, using the germline as reference.

### Cell lines and culture conditions

Jurkat, Hut-78, Hut-102 and H9 cells were purchased from ATCC (Manassas, VA, USA). The Karpas-299 cell lines were generously provided by Dr. Fu. (University of Nebraska Medical Center, Omaha, NE, USA). All cells were grown in PRMI 1640 medium or DMEM supplemented with 10% fetal bovine serum (Gibco, Life Technology, CA, USA) and 5‰ penicillin/streptomycin (Gibco, Life Technologies, CA, USA) in a humidified atmosphere with 5% CO_2_ at 37 °C. BD Vacutainer CPT™ (BD Biosciences, San Jose, CA, USA) were used for separation of peripheral blood mononuclear cells (PBMCs) from whole blood of healthy donors. PBMCs were labeled with CD3-PC5 (Beckman Coulter, USA) for 60 min at 4  °C and washed in PBS before T lymphocytes were sorted using BD FACSAria II (355 nm, 488 nm, 633 nm).

### Lentivirus-mediated small hairpin RNA (lenti-shRNA) against ITK and selective ITK inhibitor

Lentiviral vectors containing green fluorescent protein (GFP) (shControl) or a short hairpin RNA (shRNA) sequence targeting ITK (shITK-34465: TCAGTACACCAGTTCCACAGG; shITK-34466: GCCTTATATGACTACCAAACC; shITK-34467: CTCCACACACGTCTACCAGAT) were obtained from GeneChem (Shanghai, China) [24]. Jurkat, H9, Hut-78 and Karpas-299 cells were infected with shControl or shITK at a multiplicity of infection (MOI) of 1: 50, and cultured for 72 h for use in subsequent experiments. BMS-509744 (Cat No. HY-11092) and HY-11066 (Cat No. HY-11066) are both selective ITK inhibitor, which were purchased from MedChemExpress (MCE, China).

### Cell viability assays

Cell viability was evaluated by the Cell Titer-Glo Luminescent Cell Viability Assay system (Cat No. G7572, Promega Corporation, Madison, WI, USA). Cells were harvested and seeded in 96-well plates at a density of 4 × 10^4^/ml. After 2 h of incubation, the cells were treated with different concentrations of BMS-509744 and cultured for 72 h. Next, cell titer reagent (10 μl) was added to each well, and the samples were incubated on a shaker at room temperature for 10 min. Luminescent signals were measured by an LMax II instrument (Molecular Devices, Sunnyvale, CA, USA).

### Total mRNA isolation and real-time PCR

Total RNA from cell lines was extracted using TRIzol reagent (Cat No. 15596–026, Life Technologies, Carlsbad, CA, USA) according to the manufacturer’s instructions. Reverse transcription was performed using TransScript First-Strand cDNA Synthesis SuperMix (Cat No. AT301, TransGen Biotech, Beijing, China). To analyze the expression level of ITK, real-time PCR was performed using SYBR Green Master Mix (Cat No. A6001, Promega Corporation, Madison, WI, USA). Reactions were performed in a total volume of 10 μl, including 2.0 μl of cDNA (diluted 1:100), 5 μl of SYBR Green Master Mix and 0.2 μl of each specific primer (10 nM), and run in an ABI Prism 7500 real-time PCR system (Applied Biosystems, Foster City, CA, USA). The primers were as follows: ITK sense TGGTGCACAAACCTCAACCT, antisense CACATGGTTCTCCACCGTCA; GAPDH sense GCACCGTCAAGGCTGAGAAC, antisense TGGTGAAGACGCCAGTGGA; CXCR4 sense GGAGGGGATCAGTATATACA, antisense GAAGATGATGGAGTAGATGG. The cycling conditions were set at 95 °C for 10 min, followed by 45 cycles at 95 °C for 15 s and 60 °C for 1 min. GAPDH was measured for normalization. The ΔΔCt method was used to assess the relative quantifications.

### Western blot

Cells were harvested and lysed in radioimmunoprecipitation assay buffer (Cat No. 9806, Cell Signaling Technology, Danvers, MA, USA) supplemented with protease/phosphatase inhibitor cocktail (Cat No. 04693124001/04906845001, Roche, Mannheim, Germany). Protein concentrations were quantified using a Thermo BCA assay kit (Cat No. 23223, Thermo Scientific, MA, USA). Whole, cytoplasmic or nuclear cell extracts of cells were extracted and evaluated using 10% SDS-polyacrylamide gel electrophoresis or a 4–12% separating gel. After electrophoresis, the proteins were transferred to polyvinylidene fluoride membranes (Cat No. IPVH00010, Millipore Corporation, Billerica, MA, USA). The membranes were subsequently blocked with 5% defatted bovine serum albumin powder for 2 h at room temperature and then incubated with the indicated antibodies. The signals were detected using a chemiluminescence detection system (Alpha Innotech, San Leandro, CA, USA). Antibodies against ITK (Cat No. ab32039) and GAPDH (Cat No. ab9485) were provided by Abcam (Cambridge, MA, USA). The antibodies against caspase-3 (Cat No. #9662), Mcl-1 (Cat No. #5453), Bcl-xl (Cat No. #2764S), Bcl-2 (Cat No. #4223), Bak (Cat No. #12105), cyclin D1 (Cat No. #12231), phospho-cdc2-Tyr 15 (Cat No. #9111), cdc2 (Cat No. #9116), phospho-PLCγ1-Tyr783 (Cat No. #14008), phospho-Akt-Ser 473 (Cat No. #9271), Akt (Cat No. #9272), phospho-p44/42 MAPK-Thr202/Tyr 204 (Erk 1/2) (Cat No. #4370), p44/42 MAPK (Erk 1/2) (Cat No. #9102), Notch1 (Cat No. #4380T), NF-κB p65 (Cat No. #8242S), Phospho-NF-κB p65 (Ser536) (Cat No. #3031S), FAK (Cat No. #3285T), Phospho-Tyr397-FAK (Cat No. #8556) and RhoA (Cat No. #2117) were obtained from Cell Signaling Technology (Danvers, MA, USA).

### Transwell invasion and migration assay

Cell invasion assay was performed using a Matrigel invasion chamber (Cat No. 354480, Corning Costar, MA, USA) and cell migration was assessed by a Transwell assay (Cat No. 3422, Corning Costar, MA, USA). Serum-free medium (500 μl) was added into the insert of the Transwell and then were placed in the incubator at 37 °C for 2 h. Next, 1 × 10^5^ cells in 200 μl of serum-free medium were added to the top chamber and 500 μl of RPMI 1640 medium containing 30% FBS was added into the lower chamber. The cells were infected with shControl or shITK (shITK-34467) at a MOI of 1: 50, and cultured for 72 h for use in the invasion and migration assay. The cells were pretreated with vehicle and different concentrations of BMS-509744 for 12 h before they were used for the Transwell invasion and migration assay. After 24 h of incubation, the cells invading into the lower chamber were collected and counted using the Cell Titer-Glo Luminescent Cell Viability Assay system.

### Assessment of apoptosis and the cell cycle

To determine the effects of BMS-509744 on apoptosis, the FITC Annexin V/propidium iodide (PI) double staining was evaluated in the cells using the FITC Annexin V Apoptosis Detection Kit (Dojindo Laboratories, Kumamoto, Japan). Briefly, cells (2 × 10^5^) were seeded into 6-well plates and incubated for 24 h at 37 °C in culture medium containing BMS-509744 at the indicated final concentrations. After incubation, the cells were washed with PBS twice and resuspended in 100 μl of binding buffer. The cells were stained for 15 min with Annexin V-FITC and PI, and then measured by flow cytometry (BD Biosciences, San Jose, CA, USA). Approximately 1 × 10^4^ counts were made for each sample. The percentage distribution of normal, early apoptotic, late apoptotic and necrotic cells was analyzed by a BD flow cytometer.

Cell cycle progression was determined by PI (Sigm-Aldrich, St. Louis, MO, USA) staining using a flow cytometer. The cells were seeded in 6-well culture plates and cultured with BMS-509744 for 24 h. Next, the cells were fixed with 70% cold ethanol at 4 °C overnight. Then, the cells were rehydrated and washed twice with PBS, and incubated with 100 μg/ml RNase (Cat No. GE101, TransGen Biotech, Beijing, China) at 37 °C for 30 min. Then, the cell cycle was monitored by using PI staining of nuclei at room temperature for 30 min. The cell cycle was calculated using ModFit LT software (Verity Software House, Topsham, ME, USA).

### H9-cell-derived xenograft model

Six- to eight-week-old severe combined immunodeficiency (SCID) female mice were used to establish the H9-cell-derived xenograft model. H9 tumor cells (5 × 10^6^) in 0.1 ml of PBS medium with Matrigel (1: 1 ratio) were inoculated subcutaneously into the area under the right flank of each mouse. Tumor growth was measured twice weekly using a Vernier caliper. Treatments were initiated when the tumor volume reached approximately 100–150 mm^3^. The mice were randomized into four groups (n = 8/group): vehicle control, BMS-509744 (10 mg/kg), BMS-509744 (25 mg/kg) and BMS-509744 (40 mg/kg). Treatments were administered by intraperitoneal injection daily. The tumors were measured every 3 days, and tumor volume was calculated using the following formula: volume = length × width^2^/2. The treatment lasted 14 days, after which the mice were sacrificed, and the tumors were fixed in 10% formalin for histological and immunohistological analysis. All animal care and experimental procedures were performed in accordance with the Animal Care Ethics guidelines approved by the Medical Ethics Committee of the Beijing Cancer Hospital and Institute.

### In situ apoptosis detection by TUNEL staining

To detect DNA fragmentation and consequently apoptotic cells in tumor specimens, paraffin-embedded, 4-μM-thick sections from H9 tumor samples were used to identify apoptotic cells using an in situ Cell Death Detection Kit (Cat No. 11684795910, Roche, Mannheim, Germany).

### Chemosensitivity assays

Cells were infected with shITK (shITK-34465, shITK-34466 and shITK-34467) and shControl for 12 h and then cultured for 72 h before incubated with the indicated concentrations of vincristine or doxorubicin. For the ITK inhibitor, the cell lines were plated into 96-well plates and incubated with different concentrations of BMS-509744 and the indicated concentrations of vincristine or doxorubicin for 72 h. The control cells received an equivalent concentration of PBS. Inhibition rates were tested using the Cell Titer-Glo Luminescent Cell Viability Assay system. The CalcuSyn software was used to calculate the combination index values [25].

### Statistical analysis

All experiments were repeated at least three times and representative results are shown in the figures. The results are expressed as the mean ± SD. All statistical analysis was carried out using SPSS software for Windows (version 19.0). An effect was considered statistically significant at *P* < 0.05. TCR-related protein expression levels and clinical parameters were compared using the χ^2^ test. The Kaplan–Meier method was used to construct survival curves, and the results were compared using a log-rank test. The statistical significance of differences from the controls was assessed by Student’s *t* test.

## Results

### The continuous activation of the TCR signaling pathway in AITL tumor tissue

The general characteristics of the 38 AITL (27 male and 11 female) patients in this study are summarized in Table 1. All enrolled patients had histopathologically confirmed AITL. The median age at diagnosis was 58 years (range 32–81 years). All patients were in stage 3 or stage 4 at diagnosis, and 18 (50%) patients had intermediate-to-high or high international prognostic index (IPI) scores. Twenty (54%) patients presented with B symptoms, twenty-five (69.4%) patients presented with elevated serum lactate dehydrogenase (LDH) and nineteen (59.4%) patients showed elevated β_2_-MG levels.

**Table 1:** Clinical characteristics of AITL patients and correlations with the expression of phosphorylated ITK

Clinical parameters	No.	p-ITK		*P*
		Positive	Negative	
Gender				
Male	27	21	6	
Female	11	8	3	1
Age				
≤ 60	20	17	3	
> 60	18	12	6	0.26
Stage				
III	14	10	4	
IV	22	17	5	0.712
IPI score				
0–2	18	16	2	
3–5	18	11	7	0.121
ECOG				
0–1	34	26	8	
2–3	1	0	1	0.257
Ki-67				
< 75%	31	24	7	
≥ 75%	4	2	2	0.268
Extranodal invasion				
≤ 2	12	9	3	
> 2	24	18	6	1
EBER				
Positive	28	20	8	
Negative	7	6	1	0.648
B symptoms				
Positive	20	14	6	
Negative	17	14	3	0.462
LDH				
Positive	25	18	7	
Negative	11	9	2	0.69
β2-MG				
Positive	19	13	6	
Negative	13	12	1	0.195
ESR				
Positive	17	13	4	
Negative	12	9	3	1
BM involvement				
Positive	6	6	0	
Negative	29	20	9	0.304
Clinical response				
CR	11	5	6	
PR + SD + PD	22	20	2	0.004

**Fig. 1:**
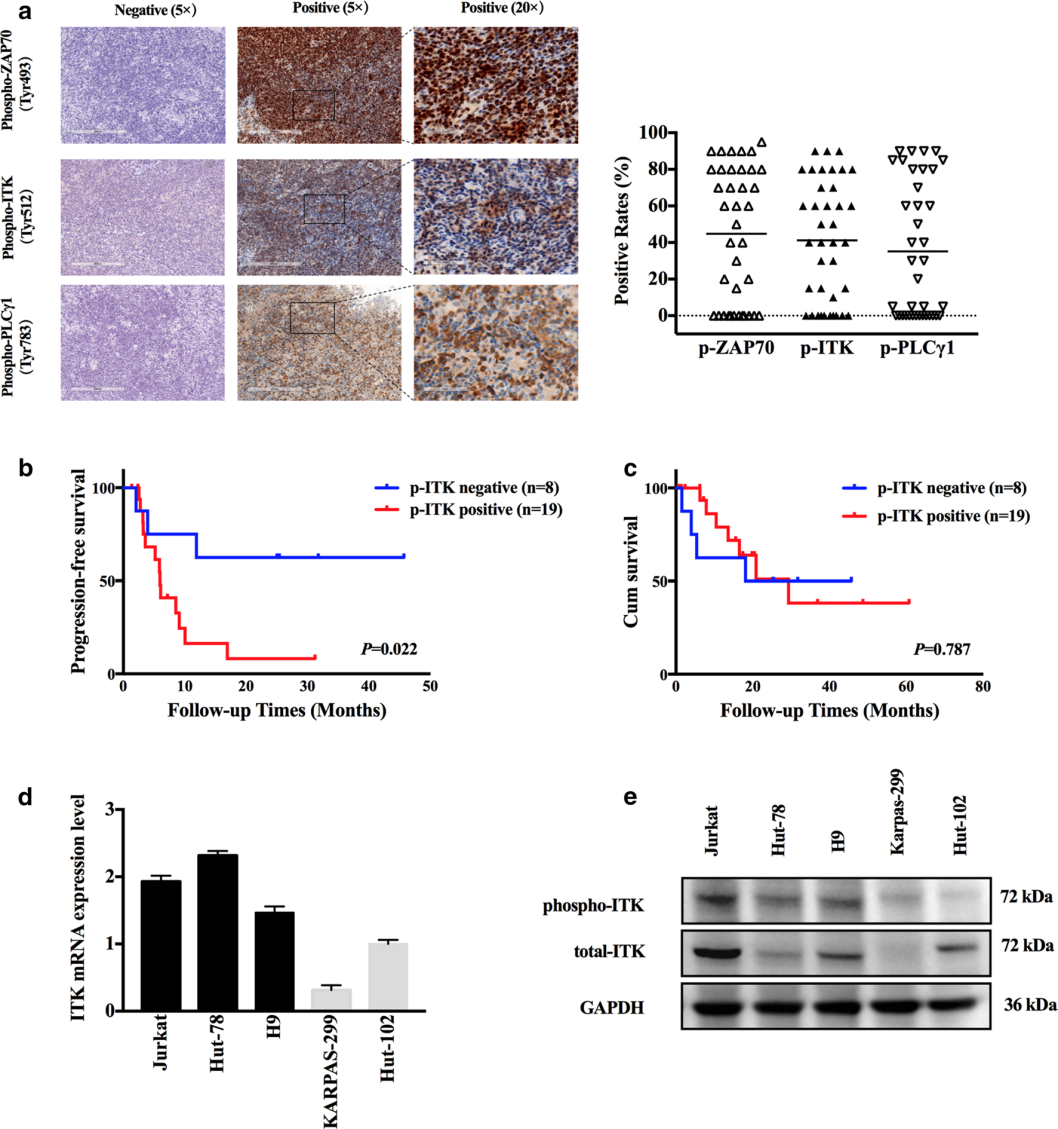
Phosphorylated ITK expression in AITL patients and malignant T cell lymphoma cell lines. **a** Immunohistochemical staining analysis of the expression of phosphorylated ZAP70, ITK and PLCγ1 in paraffin-embedded tumor tissues from AITL patients and representative pictures. Each sample was graded independently by two pathologists. **b**, **c** Kaplan–Meier curve of PFS and OS according to the expression of phosphorylated ITK. **d**, **e** ITK and phosphorylated ITK expression level in malignant T cell lymphoma cell lines were analyzed using real-time PCR and Western blot. Jurkat, Hut-78 and H9 cells expressed total ITK and phosphorylated ITK as a band at 72 kDa. GAPDH is shown as a loading control (36 kDa). Data are expressed as Mean ± SD and representative of three independent experiments

To investigate the possible role of the TCR signaling pathway in the pathogenesis of T cell lymphoid malignancies, the phosphorylation status of key regulatory tyrosine kinases was analyzed in sections of tumor tissues from 38 AITL patients. Immunohistochemical staining analysis showed that phosphorylated ZAP70, ITK and PLCγ1 were observed in 68.4%, 73.68% and 52.6% of cases, respectively (Fig. 1a). The correlations between the phosphorylation status of these kinases and clinical characteristics were evaluated in patients with complete clinical data (Additional file 2: Tables S1, Additional file 3: Table S2). Importantly, of the 33 patients who were evaluable for the response to first-line therapy, lower CR rates were observed in patients positive for phospho-ITK expression than in patients negative for phospho-ITK expression (20% vs. 75%, *P *= 0.004, Table 1). Furthermore, survival analysis was performed in twenty-seven patients who did not undergo SCT after the first-line chemotherapy. After a median follow-up time of 16.53 months (range 0.6–60.67 months), 16 (59.3%) patients relapsed or progressed and 11 (40.7%) patients died. Patients with negative expression of phosphorylated ITK had a median PFS of 25.1 months and those with positive expression of phosphorylated ITK had a median PFS of 5.17 months (*P *= 0.022). The median OS of the phospho-ITK negative group and the phospho-ITK positive group were 21.65 months and 15.53 months (*P *= 0.787), respectively (Fig. 1b, c). These data suggested that the intracellular TCR signaling pathway, especially ITK, may play an essential role in the pathogenesis of T cell malignancies.

Next, patients with fresh frozen tumor tissues and matched peripheral blood samples were subjected to WES (n = 12). The epigenetic related gene *TET2* was the most frequently mutated gene among those analyzed (8/12; 67%). The *RHOA* G17 V variant was identified in 5 of 12 patients (41.7%) (Table 2). Apart from *RHOA* and *TET2* mutations, a high frequency of diverse mutations in TCR signaling-related genes were also present in AITL tumor samples (11/12, 91.7%). The somatic TCR signaling related mutations were frequently observed in these patients, such as *CD28*, *FYN*, *CARD11*, *LCK*, *KRAS*, *PIK3R1*, *VAV1*, *ITK, ZAP70* and *AKT1*. These results also suggested that the TCR signaling pathway play an essential role in the pathogenesis of T-cell lymphoid malignancies.

**Table 2:** Characteristics of variants identified by whole exome sequencing of TCR-related genes in 12 AITL samples

Gene	Amino acid change	Mutation type	Domain	Frequency (%)
RHOA	G17V	Missense	GTP binding	41.7
ZAP70	K333R, A601V, R175H, A76E, A475T	Missense	SH2, protein kinase	41.7
CARD11	A704V, E373Q, S904I, A701V	Missense	PDZ	33.3
CD28	D124E, T195P, R198H	Missense	Extracellular, cytoplasmic	25
FYN	T15M, D390G, A412V	Missense	SH2, protein kinase	25
LCK	R168Q, A305T, A349T, Q504H	Missense	SH2, protein kinase	33.3
PIK3CA	W824C, K1063R, Q859E, L94P	Missense		41.7
ITK	A397D, A525T	Missense	Protein kinase	16.7
KRAS	G13C, A146S	Missense	GTP binding	16.7
PIK3R1	A48V, K567E, A682V	Missense	SH3, SH2	25
VAV1	C7Y, L325P	Missense		16.7
AKT1	A409V	Missense	Protein kinase	8.3

### ITK inhibition inhibits the proliferation through the suppression of the TCR-dependent signaling pathway

**Fig. 2:**
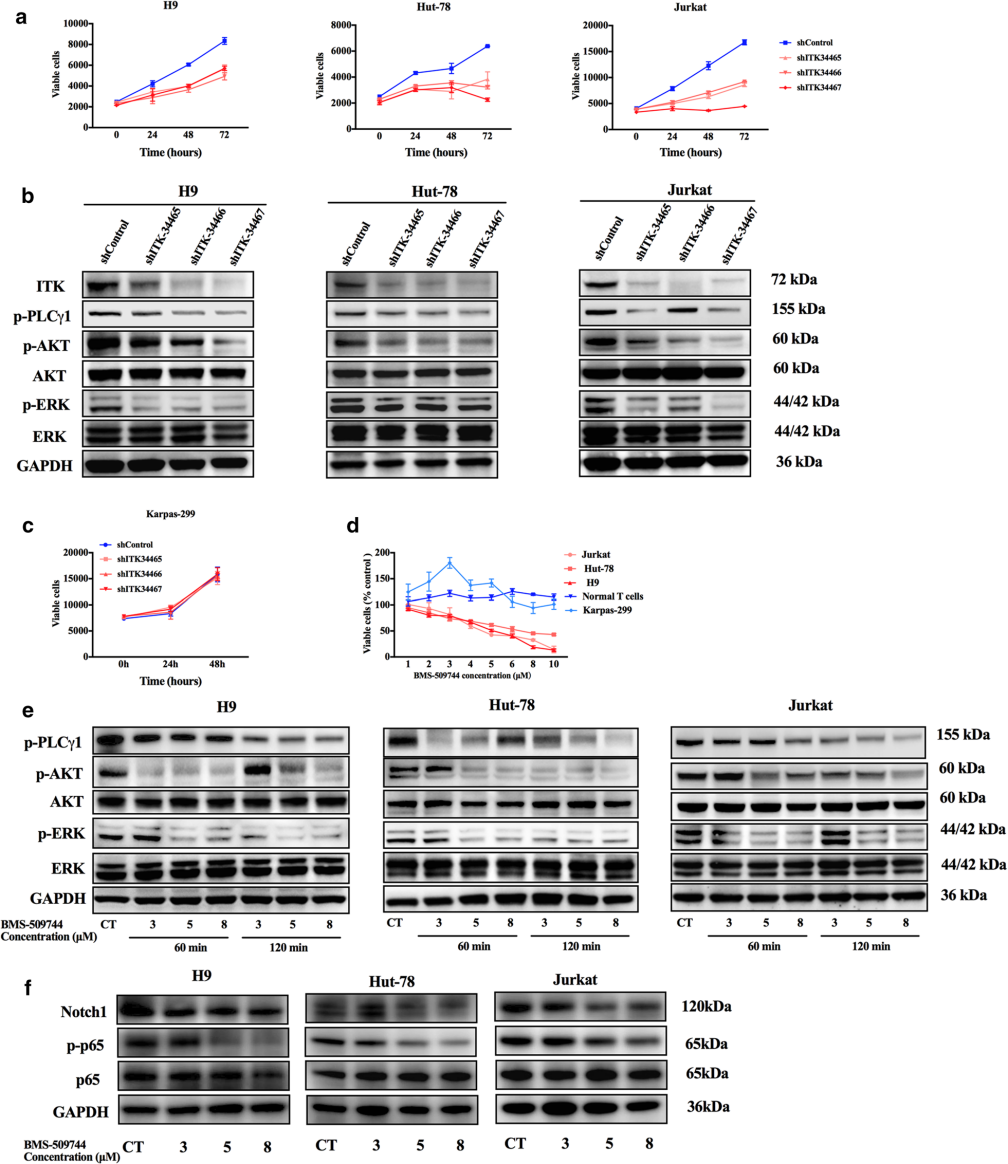
ITK inhibition reduced proliferation and inhibited TCR signaling in malignant T cells. **a** Jurkat, Hut-78, and H9 cells were transfected with vectors carrying ITK shRNA (shITK-34465, shITK-34466 and shITK-34467) or a nonspecific shRNA (shControl). Viable cells were analyzed using the Cell Titer-Glo Luminescent Cell Viability Assay. **b** Jurkat, Hut-78, and H9 cells transfected with shITK or shControl were subjected to Western blotting analysis of p-PLCγ1, p-Akt, Akt, p-Erk, and Erk. GAPDH is shown as a loading control. **c** Karpas-299 cells were transfected with carrying ITK shRNA (shITK-34465, shITK-34466 and shITK-34467) or a nonspecific shRNA (shControl). Viable cells were calculated using the Cell Titer-Glo Luminescent Cell Viability Assay. **d** Jurkat, Hut-78, H9, Karpas-299 and T cells from healthy donors were treated with the indicated concentrations of the ITK inhibitor BMS-509744. Viable cells (% control) were calculated. **e** Jurkat, Hut-78, and H9 cells were pretreated with BMS-509744 (3 μM, 5 μM, or 8 μM) for the indicated times (60 min, or 90 min), and the whole-cell extracts were subjected to Western blot. **f** Jurkat, Hut-78, and H9 cells were pretreated with BMS-509744 (3 μM, 5 μM, or 8 μM) for 24 h, and the whole-cell extracts were subjected to Western blot. Data are expressed as Mean ± SD and representative of three independent experiments. Data are expressed as Mean ± SD and representative of three independent experiments

To further elucidate the essential role of ITK in the pathogenesis of AITL, several malignant T-cell leukemia or lymphoma cell lines (Jurkat, Hut-78, Hut-102, Karpas-299 and H9) were selected for in vitro functional assays. Western Blotting and Real-time PCR analysis demonstrated that TCR signaling key regulatory kinase ITK is highly activated in H9, Hut-78 and Jurkat cell lines accompanied with ITK protein overexpression. Karpas-299 cell line, which had low expression of protein ITK and phospho-ITK, was used as a negative control. (Figure 1d, e). To analyze the functional role of ITK, the malignant T cells were transfected with lentiviral particles carrying a shRNA expression vector designed to knock down ITK expression (shITK-34465, shITK-34466 and shITK-34467). A lentiviral vector containing a nonspecific shRNA sequence was used as a negative control. The knockdown efficacy of the three individual ITK shRNAs in malignant T cell lymphoma cell lines was confirmed by Western blotting analysis (Fig. 2b). Then, the cell proliferation of shControl and shITK transfected cells was compared using the Cell Titer-Glo Luminescent Cell Viability Assay system. As shown in Fig. 2a, the reduction of ITK expression inhibited the proliferation of H9 cells. The inhibition rates of shITK-34465, shITK-34466 and shITK-34467 were 32%, 41% and 32% relative to the shControl cells, respectively. Similar results were also observed in Jurkat and Hut-78 cells, which indicated that ITK expression is critical for the growth of malignant T cell lymphoma cells. In contrast, the reduction of ITK expression did not influence the proliferation of Karpas-299 cells (Fig. 2c). More importantly, the selective ITK inhibitor BMS-509744 and HY-11066 also suppressed the proliferation of these malignant T cells (Fig. 2d; Additional file 1: Figure S1). But the viability of normal T cells from healthy donors and negative control cell line Karpas-299 were not affected after incubation with the indicated concentrations of ITK inhibitor BMS-509744 (Fig. 2d).

To clarify the possible impacts of ITK inhibition on the TCR signaling pathway, the related cascades were next investigated by immunoblotting analysis. Compared with those transfected with the shControl vector, H9 cells transfected with shITK exhibited decreased ITK expression and reduced phosphorylation of PLCγ-1, ERK1/2, and AKT. Similar results occurred in Jurkat and Hut-78 cells (Fig. 2b). More importantly, the ITK inhibitor BMS-509744 also substantially suppressed the phosphorylation of downstream TCR cascades (PLCγ-1, ERK and Akt) after 60 min and 120 min of treatment (Fig. 2e). Thus, ITK inhibition could suppress the proliferation of malignant T cells by downregulating the transduction and activation of the TCR signaling pathway.

To further investigate the other related pathways, western blotting analysis was used to detect the activity of Notch1 and phosphorylation of NF-κB (p-p65) after the cells were incubated with the indicated concentrations of BMS-509744 for 24 h (Fig. 2f). Results from this analysis revealed that inhibition of ITK could also downregulate the expression of Notch1 and phospho-NF-κB.

### ITK inhibition reduces the invasion and migration of malignant T cell lymphoma

**Fig. 3:**
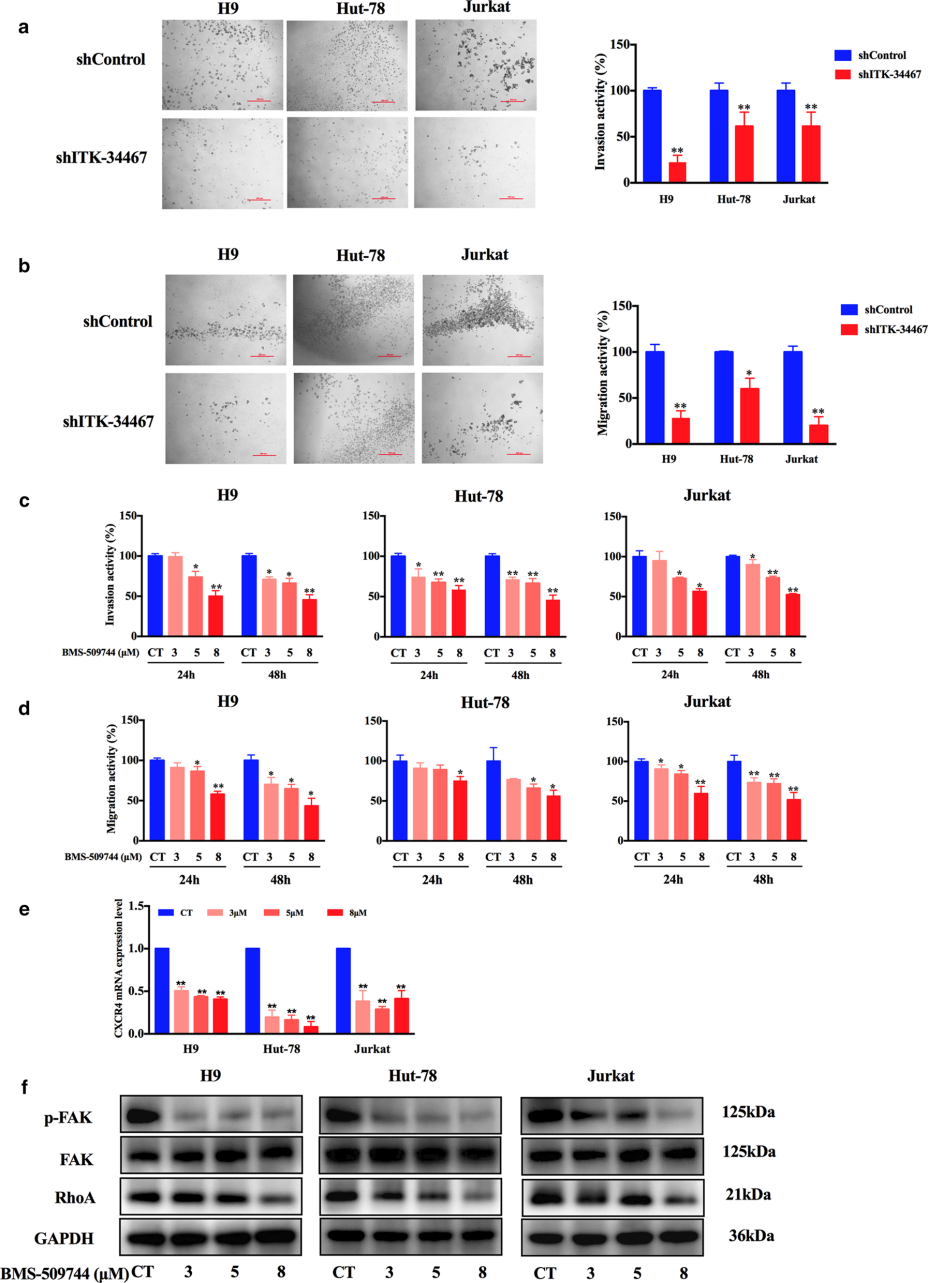
ITK inhibition reduced invasion and migration in malignant T cell lymphoma cell lines. **a**, **c** Jurkat, Hut-78 and H9 cells transfected with vectors carrying ITK shRNA (shITK-34467) or shControl or pretreated with BMS-509744 (3 μM, 5 μM, or 8 μM) were subjected to invasion assays in Transwell chambers coated with Matrigel. **b**, **d** Jurkat, Hut-78, and H9 cells transfected with vectors carrying ITK shRNA (shITK-34467) or shControl or pretreated with BMS-509744 (3 μM, 5 μM, or 8 μM) were subjected to migration assays using Transwell chambers. The cells were monitored by a CellTiter-Glo Luminescent Cell Viability Assay. Scale bar 200 μm. **e** H9, Hut-78 and Jurkat cells were treated with different concentrations of BMS-509744 (3 μM, 5 μM, or 8 μM) for 24 h. CXCR4 expression level were analyzed using real-time PCR. **f** Cells were treated with indicated concentrations of BMS-509744 for 24 h, then whole cell lysates were detected by western blot for phosphorylated FAK and total RhoA. GAPDH is shown as a loading control. Data are expressed as Mean ± SD and representative of three independent experiments. Statistical analysis was performed using Student’s *t* test. *P < 0.05, **P < 0.001 compared with the control group

Previous studies have reported that ITK may regulate the migration of T cells [26, 27]. Next, the possible effect of ITK inhibition on the motility of malignant T cells was measured in the Transwell assay system. Similar to the inhibitory effect on tumor cell proliferation, compared with the cells transfected with the shControl vector, the invasion and migration activity of these malignant T cells was decreased after shITK vector transfection (Fig. 3a, b). Consistent with these results, treatment with the ITK inhibitor BMS-509744 also efficiently inhibited invasion and migration in malignant T cells in a dose- and time-dependent manner (Fig. 3c, d; Additional file 1: Figure S2B). These data demonstrate that the activation of ITK essentially regulated the motility of these neoplastic cells. As a negative control, the effect on the invasion and migration activity of Karpas-299 was not affected after shITK vector transfection (Additional file 1: Figure S2A).

To determine the molecular mechanism by which ITK inhibitor suppressed cell migration and invasion, we investigated whether ITK inhibitor could modulate motility via C-X-C receptor 4 (CXCR4), focal adhesion kinase (FAK) and RhoA, which is involved in migration and invasion of malignant lymphoma cells [28, 29]. For this purpose, we treated malignant T cell lymphoma cells with BMS-509744 for 24 h and then conducted q-PCR and Western blotting analysis. The results demonstrated that ITK inhibition significantly decreased the expression of CXCR4 and RhoA and the activation of FAK compared with the vehicle group (Fig. 3e, f).

### ITK inhibition induces apoptosis and blocks cell cycle progression at the G2 phase in malignant T cells

**Fig. 4:**
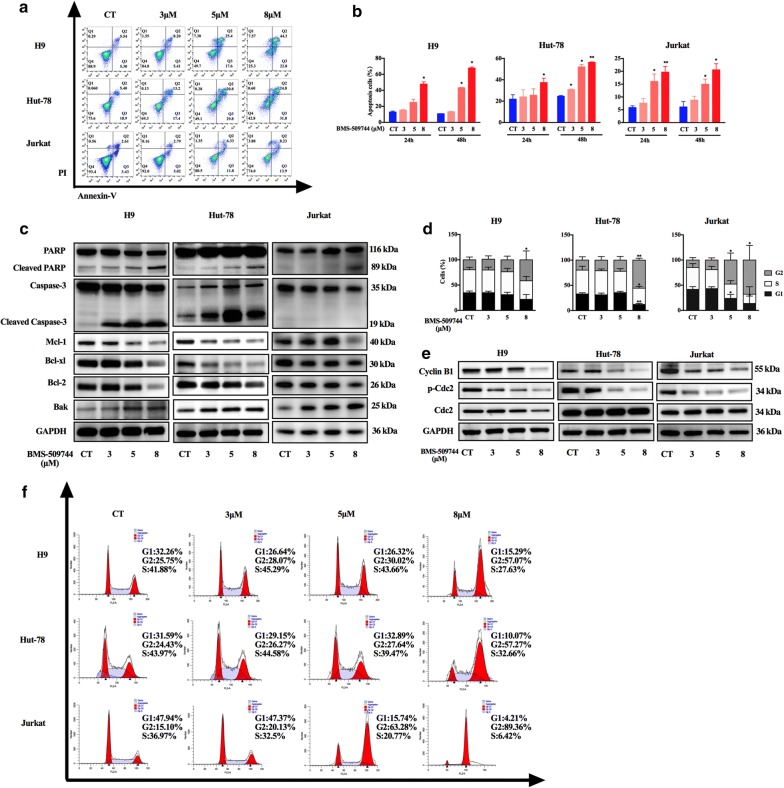
ITK inhibition induced apoptosis and G2/M phase arrest in malignant T cell lymphoma cells. **a**, **b** Jurkat, Hut-78 and H9 cells (2 × 10^5^) were treated with BMS-509744 (3 μM, 5 μM, or 8 μM) for 24 and 48 h, and apoptotic cells were quantified using flow cytometry. **c** Hut-78, H9 and Jurkat cells (2 × 10^5^) were treated with BMS-509744 (3 μM, 5 μM, or 8 μM) for 48 h. Whole-cell extracts were subjected to Western blot using antibodies directed against caspase-3, PARP, Mcl-1, Bcl-xl, Bcl-2, and Bak. GAPDH is shown as a loading control. **d**, **f** Jurkat, Hut-78 and H9 cells (2 × 10^5^) were treated with different concentrations of BMS-509744 (3 μM, 5 μM, or 8 μM) for 24 h, and the cell cycle profiles of the populations were measured using flow cytometry. **e** Western blot was used to quantify the expression of cell cycle-related proteins (p-cdc2, cdc2 and cyclin B1) after treatment with different concentrations of BMS-509744 (3 μM, 5 μM, or 8 μM) for 48 h. GAPDH is shown as a loading control. Data are expressed as Mean ± SD and representative of three independent experiments. Statistical analysis was performed using Student’s *t* test. *P < 0.05, **P < 0.001 compared with control group

To further investigate the mechanisms by which ITK inhibition induced cytotoxic effects on T cell lymphoma, the proportion of apoptotic cells was analyzed following treatment with the ITK inhibitor BMS-509744. As shown in Fig. 4a, b, the percentages of apoptotic H9 cells were 15.25 ± 0.8%, 25 ± 3.8% and 47.9 ± 2.7% upon treatment with three increasing doses of the ITK inhibitor for 24 h, suggesting that ITK inhibition mediated dose- and time-dependent apoptotic cell death in malignant T cell lines. Similar effects were also observed in Hut-78 cells. However, the apoptotic cells induced by the ITK inhibitor was not that significant in Jurkat cells compared with H9 and Hut-78. Furthermore, Western blotting analysis revealed that ITK inhibitor treatment resulted in an increase in the expression of the active forms of caspase-3 and poly-ADP ribose poly-merase (PARP). In addition, expression of the anti-apoptotic proteins, Bcl-2, Mcl-1, and Bcl-xl, was noticeably decreased in response to ITK inhibition, while expression of the pro-apoptotic protein Bak was increased (Fig. 4c). These results demonstrate that ITK inhibition promotes the induction of apoptosis via regulation of the intrinsic mitochondrial apoptosis pathway.

Next, cell cycle arrest was assayed by flow cytometry to further characterize the mechanism underlying this anti-proliferative activity. As shown in Fig. 4d, f, exposure of H9 cells to three increasing doses of the ITK inhibitor increased the percentage of cells in the G2 phase from 19.7 ± 5.4% in the control group to 21.4 ± 6.5%, 23.7 ± 7.3%, and 41.5 ± 17.4% in the treatment groups. Similar results were also observed in Jurkat and Hut-78 cells. The G2/M transition-related cell cycle proteins cyclinB1 and phosphorylated cdc2 were decreased in ITK inhibitor-treated tumor cells (Fig. 4e). These data indicate that ITK inhibition suppressed the proliferation of tumor cells by inducing cell cycle arrest at the G2/M checkpoint. As the negative control cell line, the BMS-509744 did not induce apoptosis and cell cycle arrest in Karpas-299 cells (Additional file 1: Figure S4a, b).

### ITK inhibition suppressed tumor growth in a H9-derived xenograft model

**Fig. 5:**
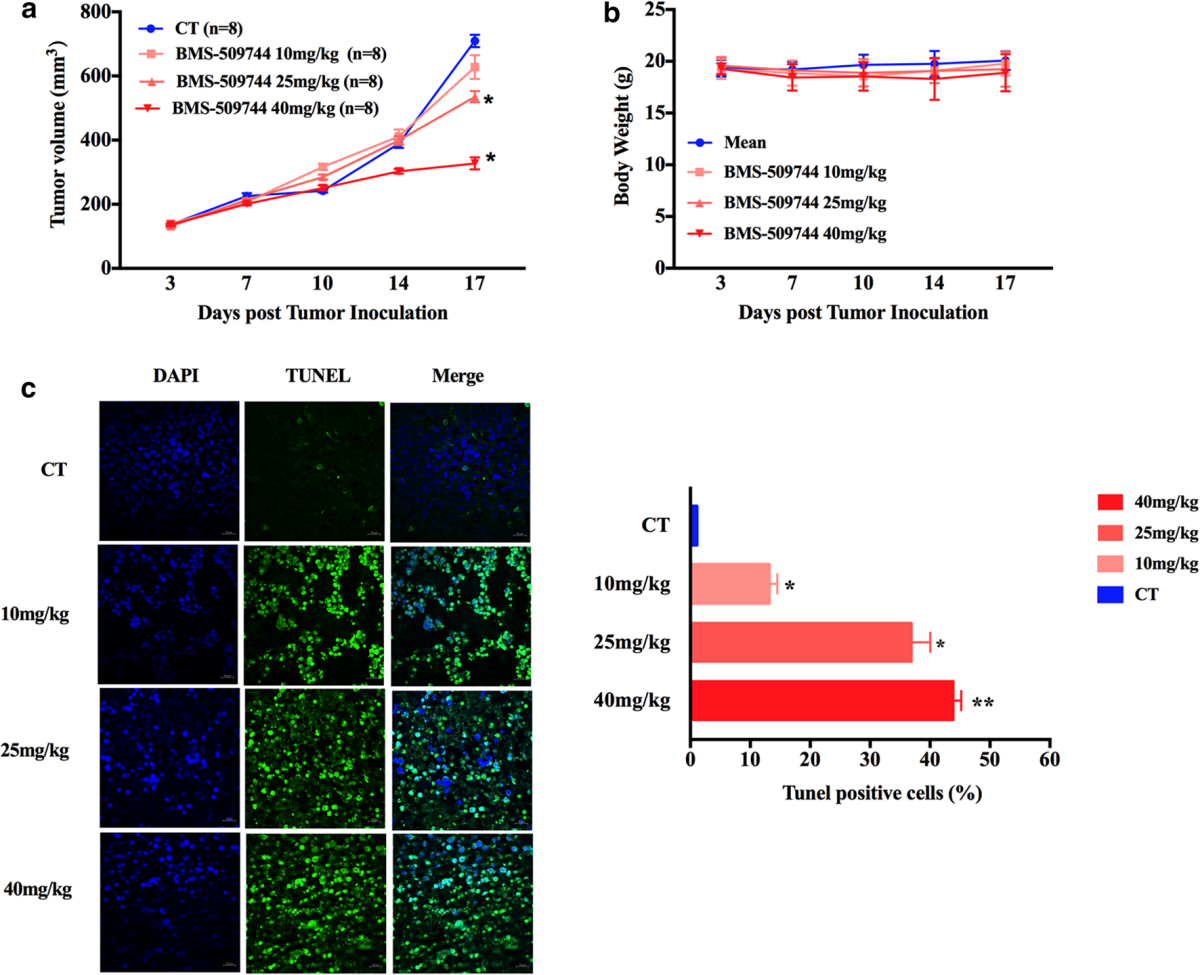
BMS-509744 treatment inhibited tumor growth in a mouse xenograft model of malignant T cell lymphoma. **a**, **b** SCID mice were inoculated subcutaneously with 5 × 10^6^ H9 cells and each group contained 8 mice. BMS-509744 (10 mg/kg, 25 mg/kg and 40 mg/kg) was administrated to the mice at the indicated concentrations once daily for 14 days when the tumor volume reached 200 mm^3^. The tumor volume of mice was measured every 3 days during the treatment. Statistical analysis was performed using Student’s *t* test. *P < 0.05, **P < 0.001 compared with control group. Data are expressed as Mean ± SEM and were derived from each treatment arm (n = 8). **c** Apoptosis of tumor tissue was assessed by a TUNEL assay. The nuclei were counterstained with DAPI. Representative images show apoptotic (fragmented) DNA (green staining) and the corresponding cell nuclei (blue) staining. Scale bar 20 μm. Data are expressed as Mean ± SEM

The in vivo antitumor activity of ITK inhibition was examined in SCID mice inoculated with H9 lymphoma cells. SCID mice were inoculated with H9 cells to establish a T cell lymphoma cell-derived xenograft. The mice were randomized into four treatment groups according to tumor volume and body weight. Mice were treated with the ITK inhibitor BMS-509744 (10, 25 and 40 mg/kg) once daily for 14 days by intraperitoneal injection. As shown in Fig. 5a, the ITK inhibitor reduced tumor growth by 11.49%, 24.53% and 53.82% (*P *= 0.502, *P *= 0.033 and *P *= 0.000) in response to 10, 25 and 40 mg/kg BMS-509744 treatment, respectively. The body weight of tumor-bearing mice was not significantly affected post ITK inhibitor treatment. To further evaluate the apoptosis status of tumor after the treatment of BMS-509744, we performed TUNEL staining on paraffin sections of tumor samples collected from H9 xenografts post ITK inhibitor treatment. As shown in Fig. 5c, BMS-509744 treatment produced an increase in apoptosis compared with that in the vehicle group. These data demonstrate that the reduction in the progression of the tumor is observed at high doses of the ITK inhibitor BMS-509744 in vivo.

### ITK inhibition enhances the antitumor activity of chemotherapeutic agents in T cell lymphoma

**Fig. 6:**
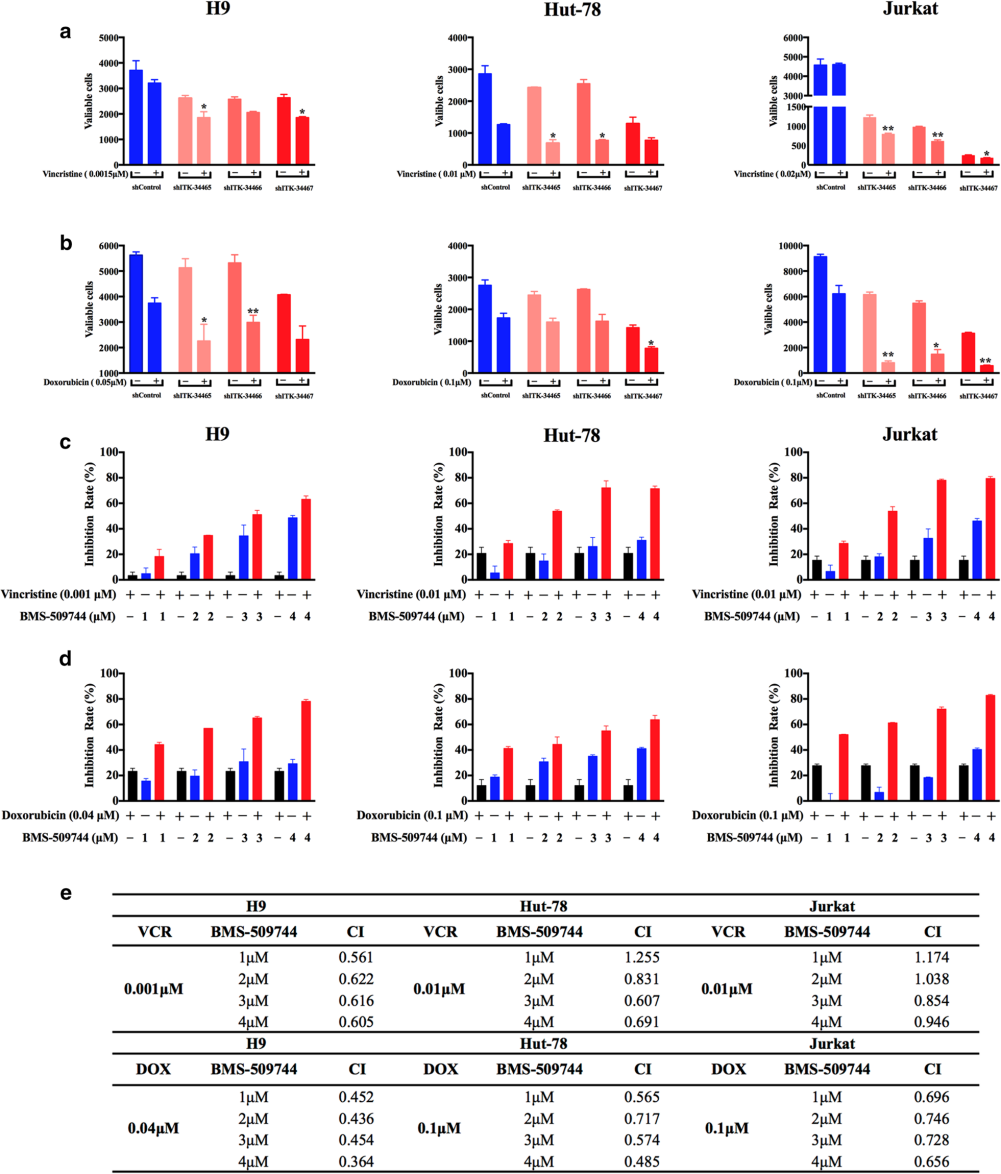
Inhibition of ITK expression enhanced chemosensitivity to common chemotherapeutic agents in malignant T cells. Tumor cells transfected with shITK (shITK-34465, shITK-34466 and shITK-34467) or shControl were exposed to vincristine (**a**) or doxorubicin (**b**) for 72 h. Cell viability was measured using a Cell Titer-Glo Luminescent Cell Viability Assay. **c** Tumor cells were seeded in opaque-walled 96-well plates at a density of 4 × 10^4^ cells/ml and incubated with varying, minimally toxic concentrations of BMS-509744 in the presence or absence of fixed concentrations of vincristine (Jurkat, 0.01 μM; Hut-78, 0.01 μM; H9, 0.001 μM) for 72 h. **d** Tumor cells were incubated with varying minimally toxic concentrations of BMS-509744, in the presence or absence of fixed concentrations of doxorubicin (Jurkat 0.1 μM, Hut-78 0.1 μM and H9 0.04 μM) for 72 h. The cell viability was measured using the Cell Titer-Glo Luminescent Cell Viability Assay. Inhibition rates were calculated by (1 − Dosing/Vehicle) × 100%. **e** The CI values at different concentrations of BMS-509744 with fixed concentrations of vincristine or doxorubicin in malignant T cells were calculated using CalcuSyn software. *VCR* vincristine, *DOX* doxorubicin. Data are expressed as Mean ± SD and representative of three independent experiments. Statistical analysis was performed using Student’s *t* test. *P < 0.05, **P < 0.001 compared with the control group

Given the observed effects of ITK knockdown on the survival and proliferation of malignant T cell lymphoma, we next investigated whether ITK suppression enhanced chemosensitivity. shITK- or shControl-transfected H9 cells were incubated with vincristine (0.0015 μM) or doxorubicin (0.05 μM) for 72 h, which are commonly used in the treatment of AITL. (Figure 6a, b). shITK transfection synergistically enhanced the antitumor effect of the vincristine (shITK-34465: 29%; shITK-34466: 20% and shITK-34467: 30%) in H9 cells compared with that of cells transfected with shControl (13.5%). Similarly, H9 ITK knockdown cells exhibited higher inhibition rates for doxorubicin (shITK-34465: 56%; shITK-34466: 44% and shITK-34467: 43%) than those of the shControl cells (33.4%). The resulting data showed that ITK knockdown synergistically enhances the antitumor activity of vincristine and doxorubicin. Similar results were also observed in Jurkat ITK knockdown cells and Hut-78 ITK knockdown cells. In contrast, ITK inhibition did not enhance chemosensitivity of Karpas-299 cells towards vincristine and doxorubicin (Additional file 1: Figure S3).

To further confirm the synergistic activity of combined ITK inhibition and chemotherapy, H9 cells were incubated with a fixed concentration of vincristine or doxorubicin and increasing concentrations of the ITK inhibitor for 72 h. The combination treatments resulted in decreased cell viability compared to that of each agent alone. Similar results were also observed in Jurkat and Hut-78 tumor cells (Fig. 6c, d). The combination index (CI) values were used to quantify these synergistic antitumor effects (Fig. 6e). The analysis of CI values revealed synergism for the combination of the ITK inhibitor and vincristine or doxorubicin, with CI values < 1. These results suggested that ITK inhibition could moderately enhance the antitumor effects of standard T cell lymphoma treatment regimens.

## Discussion

Recently, many investigations of pathogenic mechanisms in AITL identified frequent somatic mutations of the epigenetic regulation-related genes, including *TET2* [30–33], *IDH2* [31, 34] and *DNMT3A* [31, 35, 36]. In addition to frequent somatic mutations in *RHOA* [11, 31, 36, 37] and epigenetic modifiers, several studies have shown frequent alterations affecting TCR signaling-related genes, including *CD28* [38, 39], *FYN* [36, 39], *PLCγ1* [39] and *VAV1* [39] in cutaneous T-cell lymphomas, AITL and adult T cell leukemia/lymphoma. In this study, we identified a high frequency of diverse mutations in TCR signaling-related genes in Chinese patients with AITL. Although several reports showed some mutations within these regulatory kinases affected TCR-induced calcium responses [14–16], further functional experimentations and validation cohorts are still needed to explore the precise mechanism of these mutations. The varied expressions of TCR-associated proteins were previously analyzed among PTCL subtypes. In this study, the activation status of key tyrosine kinases in the TCR signaling pathway were first evaluated in AITL patients. Immunohistochemical staining analysis demonstrated that more than half of the patients exhibited phosphorylated ZAP70, ITK and PLCγ1 in tumor tissues of AITL patients, suggesting the continuous activation of the TCR signaling in AITL. These findings may offer additional clues for the pathogenic role of TCR signaling in AITL. The above evidences suggested that the activation of the TCR pathway might play a key role in PTCL pathogenesis, especially protein tyrosine kinase, which have been confirmed their pivotal roles in the TCR signaling transduction. Thus, it is important to evaluate the specific role of protein tyrosine kinase on TCR signaling and the related drugs against these candidate therapeutic targets.

Previous studies have evaluated the expression of intracellular proteins involved in TCR signaling, including ITK, in normal and neoplastic hematologic tissue samples [40, 41]. ITK turned out to be T-cell lineage-specific markers in the setting of lymphoid and myeloid precursor neoplasms but negative in B cell lymphoma. Among 38 AITL patients, 29 (73.6%) patients showed positive expression of phosphorylated ITK in the study. Analysis of correlations between the expression of phosphorylated ITK and the clinical features revealed that the lower CR rates and shorter PFS were observed in patients positive for phosphorylated ITK expression. However, no significant difference in OS were observed between patients positive for phosphorylated ITK expression and those negative for phosphorylated ITK expression. Given the small size of the cohort of patients analyzed for follow-up, further experimentations are still needed to investigate the relationship between the ITK expression and the prognosis of patients with AITL.

The t(5;9) (q33;q22) translocation was observed in PTCL-NOS patients, generating the ITK-SYK fusion tyrosine kinase [42]. ITK-SYK translocation triggers antigen-independent phosphorylation of TCR-proximal proteins, which has been confirmed to act as a powerful oncogenic driver in transgenic lymphoma mouse models [12]. Recent studies have demonstrated that Syk protein is aberrantly expressed in the majority of PTCLs and inhibition of Syk induces apoptosis and blocks proliferation in T-cell lymphoma cell lines [43, 44]. However, the activation status and functional role of ITK in T-cell lymphomas is still unclear. On the other hand, Liang et al. retrospectively investigated 35 cases of AITL from Taiwan and identified gain of ITK and SYK genes in 38% and 14% patients, respectively, which also suggested the importance of investigating the potential role of ITK in T cell lymphoma [45].

Given the success of the BTK inhibitor ibrutinib in the treatment of B cell lymphoma, the TEC family member ITK has become an appealing target for inhibiting TCR signaling, which plays an important role in T cell activation, development, differentiation, and cytokines production [15, 16]. ITK gene knockout mice displayed a decreased number of maturing thymocytes and showed impaired IL-2 production after stimulation through the TCR [46]. In our study, the genetic knockdown of ITK significantly compromised the proliferation, adhesion, invasion and migration of malignant T cells. In addition to genetic inhibition of ITK, the selective ITK inhibitor BMS-509744 also demonstrated similarly the antitumor and pro-apoptotic activities in vitro and in vivo. In contrast, the proliferation, invasion and migration activity were not affected after the genetic knockdown of ITK in Karpas-299 cells, which had a relatively low expression of protein ITK and phospho-ITK. These results suggested that ITK inhibitor might be an efficient therapeutic drug for the treatment ITK positive patients.

Previous studies have reported that ITK may regulate CXCR4-mediated migration and adhesion by altering the actin cytoskeleton [27]. Similarly, inhibition of ITK also significantly suppressed the mRNA expression level of CXCR4 in our study. More importantly, it is reported that FAK and RhoA played important role in modulating the motility of malignant lymphoma cells. FAK is a non-receptor tyrosine kinase that localizes at sites of cell adhesion to the extracellular matrix, which is known to regulate cell migration and invasion in many cancer types [47–49]. RhoA is a member of Rho GTPase family, which regulate the cytoskeleton and have important roles in cell migration [28, 29, 50]. Consistent with previous studies, inhibition of ITK also suppressed the migration and invasion of malignant T-cell lymphoma through directly inhibiting RhoA and FAK.

Previously, the ITK inhibitor BMS-509744 has been illustrated to effectively diminishes lung inflammation by blocking T cell activation [51]. Similarly, Zhong et al. also demonstrated that the highly selective covalent ITK and RLK inhibitor PRN694 showed inhibitory activity against T cell leukemia cells through the downregulation of NFAT1, JunB, PLCγ1, and IκBα [52]. TCR-CD28 signals play an essential role in stimulating the activation of NF-κB signaling pathway through mediating activation of IκB kinase and NF-κB, which contribute to the T-cell activation, proliferation and survival [53, 54]. NF- κB activation have been considered as important drivers of tumor growth and survival in B-cell lymphoma. Previous studies reported that inhibition of PI3K/AKT exhibited high potent activity in blocking NF-κB activation, which significantly increase tumor regression [55]. In our study, down-regulation of TCR signaling pathway through inhibiting the expression of ITK showed significant activity in inhibition of NF-κB (Fig. 2f). Furthermore, previous studies confirmed the critical role of activated NOTCH1 in the molecular pathogenesis of human T-ALL through increasing nuclear Notch1 level [56]. In this paper, western blotting exhibited that ITK inhibitor could also downregulate the activity of Notch1 level in malignant T-cell lymphoma cell lines (Fig. 2f). Further experiments are still needed to investigate connections between activating TCR signaling pathway and other T-cell related signaling pathways, which could deepen the knowledge in the molecular pathogenesis of T-cell hematopoietic malignancies.

Furthermore, the BTK/ITK inhibitor ibrutinib efficiently limits the IL-4-producing Th2 cell population and inhibits cytokine production and direct signaling to induce the activation of tumor cells [57]. However, Dondor et al. reported that BMS-509744 does not affect cell viability in HH and Jurkat cells. Due to the low expression level of ITK, the HH cell lines did not respond to BMS-509744 treatment. In our present study, BMS-509744 treatment also did not induce significant apoptosis in Jurkat cells. But we found that the cell death induced by BMS-509744 mainly through blocking cell cycle progression at the G2 phase. Thus, ITK inhibition induced cytotoxic effects on T-cell lymphoma cells through inducing the apoptosis or blocking cell cycle progression. All these results supporting the role of the key TCR signaling regulatory tyrosine kinase ITK suggested a therapeutic target for the treatment of patients with ITK-expressing T cell lymphomas.

Many studies have reported that targeted cancer therapy enhances the drug efficacy of conventional chemotherapy in the treatment of leukemia and lymphoma patients [58, 59]. Since the inhibition of TCR signaling had been demonstrated to overcome the resistance to chemotherapy in T cell lymphomas [60], the antitumor effects of the ITK inhibitor and conventional chemotherapies were further investigated in our experiment. The combination of the ITK inhibitor BMS-509744 and conventional chemotherapies, such as vincristine or doxorubicin, acted synergistically to inhibit the proliferation of malignant T cells, indicating that combining ITK inhibition with CHOP may be a promising therapeutic regimen for the treatment of AITL.

## Conclusion

In summary, our data provide the evidence of a functional role for ITK in survival, growth and motility of malignant T cells. Given the small size of the cohort of AITL patients, our findings may suggest a candidate therapeutic target and combination regimen for AITL patients, which currently lack good treatment options.

## Additional files

**Additional file 1: Figure S1.** ITK inhibitor HY-11066 inhibits the proliferation of malignant T-cell lymphoma cell lines. Jurkat, Hut-78 and H9 cells were treated with the indicated concentrations of the ITK inhibitor HY-11066. The cell viability was measured using the Cell Titer-Glo Luminescent Cell Viability Assay. Viable cells (% control) were calculated. Data are expressed as Mean ± SD and representative of three independent experiments. **Figure S2.** Transwell invasion and migration assays of ITK inhibition in Karpas-299 cells. (A) Karpas-299 cells transfected with vectors carrying ITK shRNA (shITK-34467) or shControl were subjected to invasion assays and migration assays using Transwell chambers coated with Matrigel and Transwell chambers without Matrigel respectively. The cells were monitored by a CellTiter-Glo Luminescent Cell Viability Assay. Data are expressed as Mean ± SD and representative of three independent experiments. Statistical analysis was performed using Student’s t test. *P < 0.05, **P < 0.001 compared with the control group. (B) Migration and invasion assay images of Fig. 3c, d. **Figure S3.** The chemosensitivity to common chemotherapeutic agents in Karpas-299 cells after the inhibition of ITK. Karpas-299 cells transfected with shITK (shITK-34467) or shControl were exposed to vincristine (A) or doxorubicin (B) for 72 h. Cell viability was measured using a Cell Titer-Glo Luminescent Cell Viability Assay. Data are expressed as Mean ± SD and representative of three independent experiments. Statistical analysis was performed using Student’s t test. *P < 0.05, **P < 0.001 compared with the control group. **Figure S4.** ITK inhibitor BMS-509744 have no effect on the apoptosis and cell cycle arrest in karpas-299 cells. (A) Karpas-299 cells (2 × 10^5^) were treated with BMS-509744 (3 μM, 5 μM, or 8 μM) for 24 and 48 h, and apoptotic cells were quantified using flow cytometry. (B) Karpas-299 cells (2 × 10^5^) were treated with different concentrations of BMS-509744 (3 μM, 5 μM, or 8 μM) for 24 h, and the cell cycle profiles of the populations were measured using flow cytometry. Data are expressed as Mean ± SD and representative of three independent experiments. Statistical analysis was performed using Student’s t test. *P < 0.05, **P < 0.001 compared with the control group.

**Additional file 2: Table S1.** Patients’ characteristics and correlations with the expression of p-ZAP70.

**Additional file 3: Table S2.** Patients’ characteristics and correlations with the expression of p-PLCγ1.

## Abbreviations

AITL: angioimmunoblastic T cell lymphoma
PTCL: peripheral T cell lymphoma
TCR: T-cell receptor
BCR: B-cell receptor
MHC: major histocompatibility complex
SCT: stem-cell transplantation
ITAM: immunoreceptor tyrosine-based activation motif
PLCγ1: phospholipase C-γ1
PIP2: phosphatidylinositol-4,5-bisphosphate
DAG: diacylglycerol
IP3: inositol triphosphate
PI3K: phosphatidylinositol-3-kinase
CHOP: cyclophosphamide, doxorubicin, vincristine, prednisone
OS: overall survival
PFS: progression free survival
CR: complete response
PR: partical response
SD: stable disease
PD: progression disease
IHC: immunohistochemistry
shRNA: short hairpin RNA
SCID: severe combined immunodeficiency disease
IPI: international prognostic index
LDH: lactate dehydrogenase
MG: microglobulin
PARP: poly-ADP ribose poly-merase
CI: combination index
