# Supplementary figures and images for: ITK inhibition induced in vitro and in vivo anti-tumor activity through downregulating TCR signaling pathway in malignant T cell lymphoma
Source: Cancer Cell Int. 2019 Feb 14;19:32. doi: 10.1186/s12935-019-0754-9 (PMC6376795; doi:10.1186/s12935-019-0754-9)

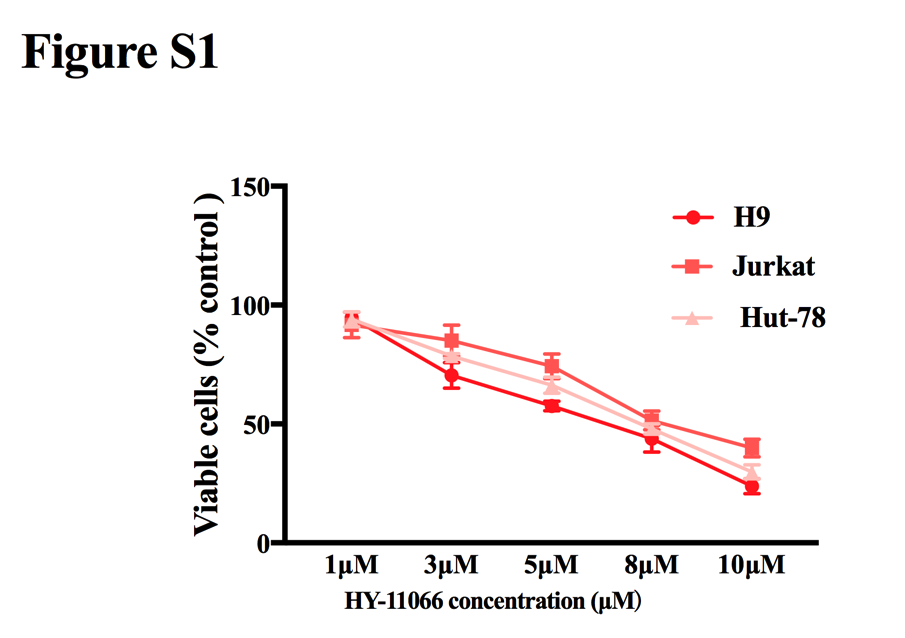

Supplement: Supplementary file 1 — Additional file 1: Figure S1. ITK inhibitor HY-11066 inhibits the proliferation of malignant T-cell lymphoma cell lines. Jurkat, Hut-78 and H9 cells were treated with the indicated concentrations of the ITK inhibitor HY-11066. The cell viability was measured using the Cell Titer-Glo Luminescent Cell Viability Assay. Viable cells (% control) were calculated. Data are expressed as Mean ± SD and representative of three independent experiments. Figure S2. Transwell invasion and migration assays of ITK inhibition in Karpas-299 cells. (A) Karpas-299 cells transfected with vectors carrying ITK shRNA (shITK-34467) or shControl were subjected to invasion assays and migration assays using Transwell chambers coated with Matrigel and Transwell chambers without Matrigel respectively. The cells were monitored by a CellTiter-Glo Luminescent Cell Viability Assay. Data are expressed as Mean ± SD and representative of three independent experiments. Statistical analysis was performed using Student’s t test. *P < 0.05, **P < 0.001 compared with the control group. (B) Migration and invasion assay images of Fig. 3c, d. Figure S3. The chemosensitivity to common chemotherapeutic agents in Karpas-299 cells after the inhibition of ITK. Karpas-299 cells transfected with shITK (shITK-34467) or shControl were exposed to vincristine (A) or doxorubicin (B) for 72 h. Cell viability was measured using a Cell Titer-Glo Luminescent Cell Viability Assay. Data are expressed as Mean ± SD and representative of three independent experiments. Statistical analysis was performed using Student’s t test. *P < 0.05, **P < 0.001 compared with the control group. Figure S4. ITK inhibitor BMS-509744 have no effect on the apoptosis and cell cycle arrest in karpas-299 cells. (A) Karpas-299 cells (2 × 10^5^) were treated with BMS-509744 (3 μM, 5 μM, or 8 μM) for 24 and 48 h, and apoptotic cells were quantified using flow cytometry. (B) Karpas-299 cells (2 × 10^5^) were treated with different concentrations of BMS-509744 ( [file 12935_2019_754_MOESM1_ESM.zip › Figure S1.tiff]

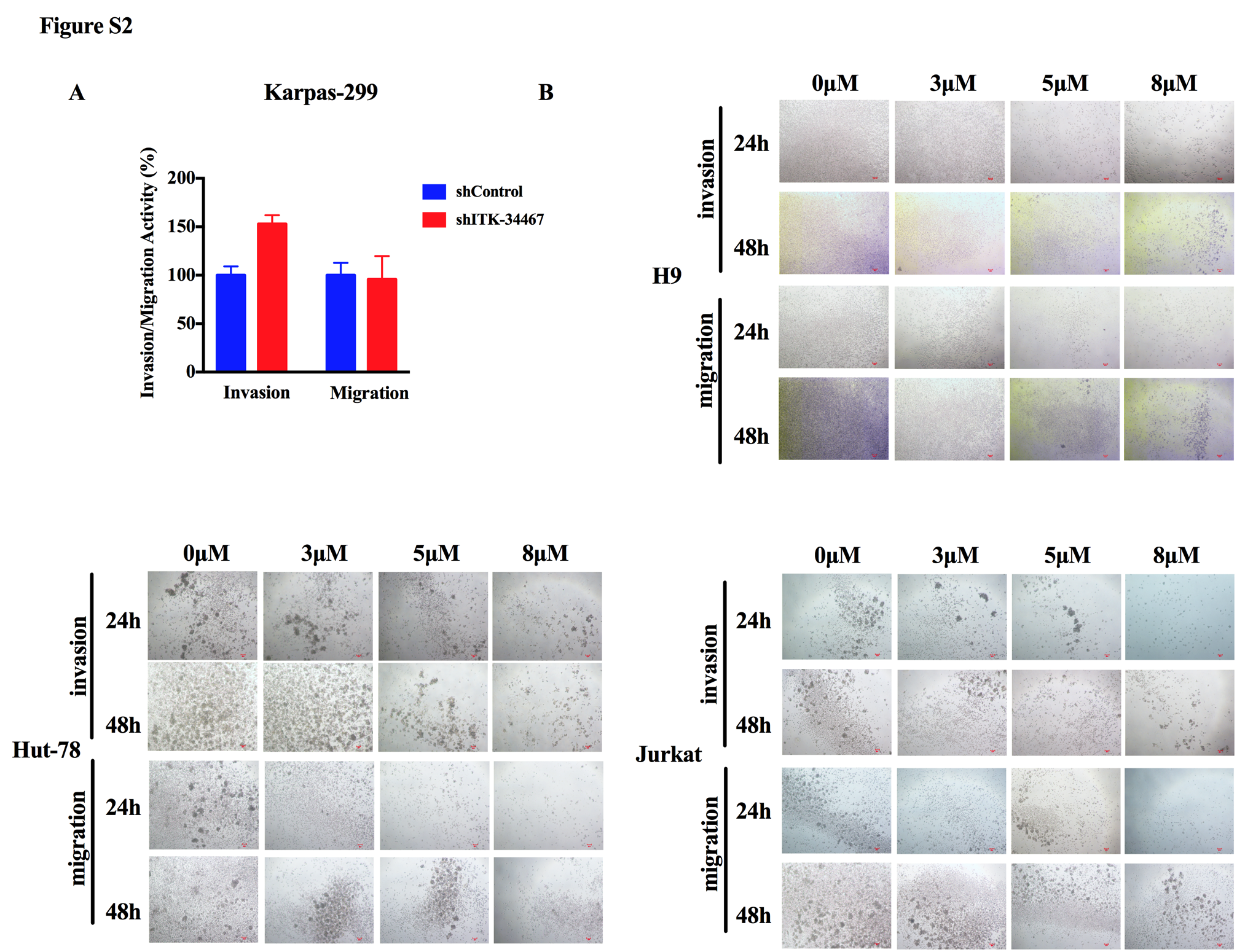

Supplement: Supplementary file 1 — Additional file 1: Figure S1. ITK inhibitor HY-11066 inhibits the proliferation of malignant T-cell lymphoma cell lines. Jurkat, Hut-78 and H9 cells were treated with the indicated concentrations of the ITK inhibitor HY-11066. The cell viability was measured using the Cell Titer-Glo Luminescent Cell Viability Assay. Viable cells (% control) were calculated. Data are expressed as Mean ± SD and representative of three independent experiments. Figure S2. Transwell invasion and migration assays of ITK inhibition in Karpas-299 cells. (A) Karpas-299 cells transfected with vectors carrying ITK shRNA (shITK-34467) or shControl were subjected to invasion assays and migration assays using Transwell chambers coated with Matrigel and Transwell chambers without Matrigel respectively. The cells were monitored by a CellTiter-Glo Luminescent Cell Viability Assay. Data are expressed as Mean ± SD and representative of three independent experiments. Statistical analysis was performed using Student’s t test. *P < 0.05, **P < 0.001 compared with the control group. (B) Migration and invasion assay images of Fig. 3c, d. Figure S3. The chemosensitivity to common chemotherapeutic agents in Karpas-299 cells after the inhibition of ITK. Karpas-299 cells transfected with shITK (shITK-34467) or shControl were exposed to vincristine (A) or doxorubicin (B) for 72 h. Cell viability was measured using a Cell Titer-Glo Luminescent Cell Viability Assay. Data are expressed as Mean ± SD and representative of three independent experiments. Statistical analysis was performed using Student’s t test. *P < 0.05, **P < 0.001 compared with the control group. Figure S4. ITK inhibitor BMS-509744 have no effect on the apoptosis and cell cycle arrest in karpas-299 cells. (A) Karpas-299 cells (2 × 10^5^) were treated with BMS-509744 (3 μM, 5 μM, or 8 μM) for 24 and 48 h, and apoptotic cells were quantified using flow cytometry. (B) Karpas-299 cells (2 × 10^5^) were treated with different concentrations of BMS-509744 ( [file 12935_2019_754_MOESM1_ESM.zip › Figure S2.tiff]

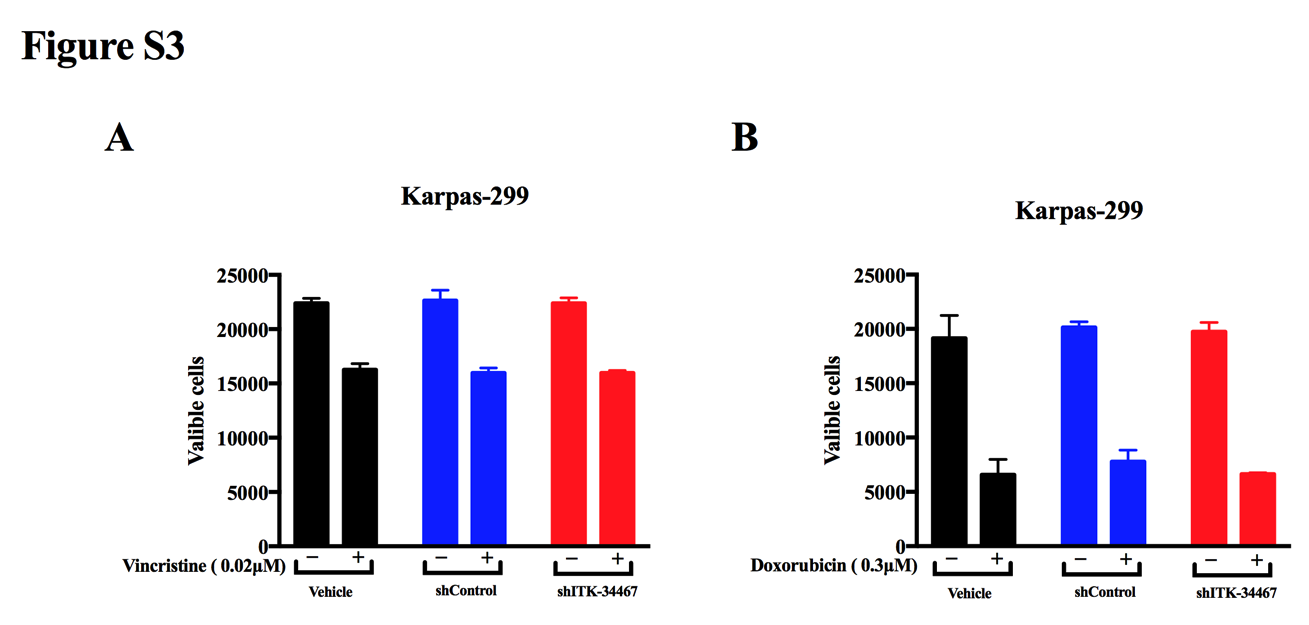

Supplement: Supplementary file 1 — Additional file 1: Figure S1. ITK inhibitor HY-11066 inhibits the proliferation of malignant T-cell lymphoma cell lines. Jurkat, Hut-78 and H9 cells were treated with the indicated concentrations of the ITK inhibitor HY-11066. The cell viability was measured using the Cell Titer-Glo Luminescent Cell Viability Assay. Viable cells (% control) were calculated. Data are expressed as Mean ± SD and representative of three independent experiments. Figure S2. Transwell invasion and migration assays of ITK inhibition in Karpas-299 cells. (A) Karpas-299 cells transfected with vectors carrying ITK shRNA (shITK-34467) or shControl were subjected to invasion assays and migration assays using Transwell chambers coated with Matrigel and Transwell chambers without Matrigel respectively. The cells were monitored by a CellTiter-Glo Luminescent Cell Viability Assay. Data are expressed as Mean ± SD and representative of three independent experiments. Statistical analysis was performed using Student’s t test. *P < 0.05, **P < 0.001 compared with the control group. (B) Migration and invasion assay images of Fig. 3c, d. Figure S3. The chemosensitivity to common chemotherapeutic agents in Karpas-299 cells after the inhibition of ITK. Karpas-299 cells transfected with shITK (shITK-34467) or shControl were exposed to vincristine (A) or doxorubicin (B) for 72 h. Cell viability was measured using a Cell Titer-Glo Luminescent Cell Viability Assay. Data are expressed as Mean ± SD and representative of three independent experiments. Statistical analysis was performed using Student’s t test. *P < 0.05, **P < 0.001 compared with the control group. Figure S4. ITK inhibitor BMS-509744 have no effect on the apoptosis and cell cycle arrest in karpas-299 cells. (A) Karpas-299 cells (2 × 10^5^) were treated with BMS-509744 (3 μM, 5 μM, or 8 μM) for 24 and 48 h, and apoptotic cells were quantified using flow cytometry. (B) Karpas-299 cells (2 × 10^5^) were treated with different concentrations of BMS-509744 ( [file 12935_2019_754_MOESM1_ESM.zip › Figure S3.tiff]

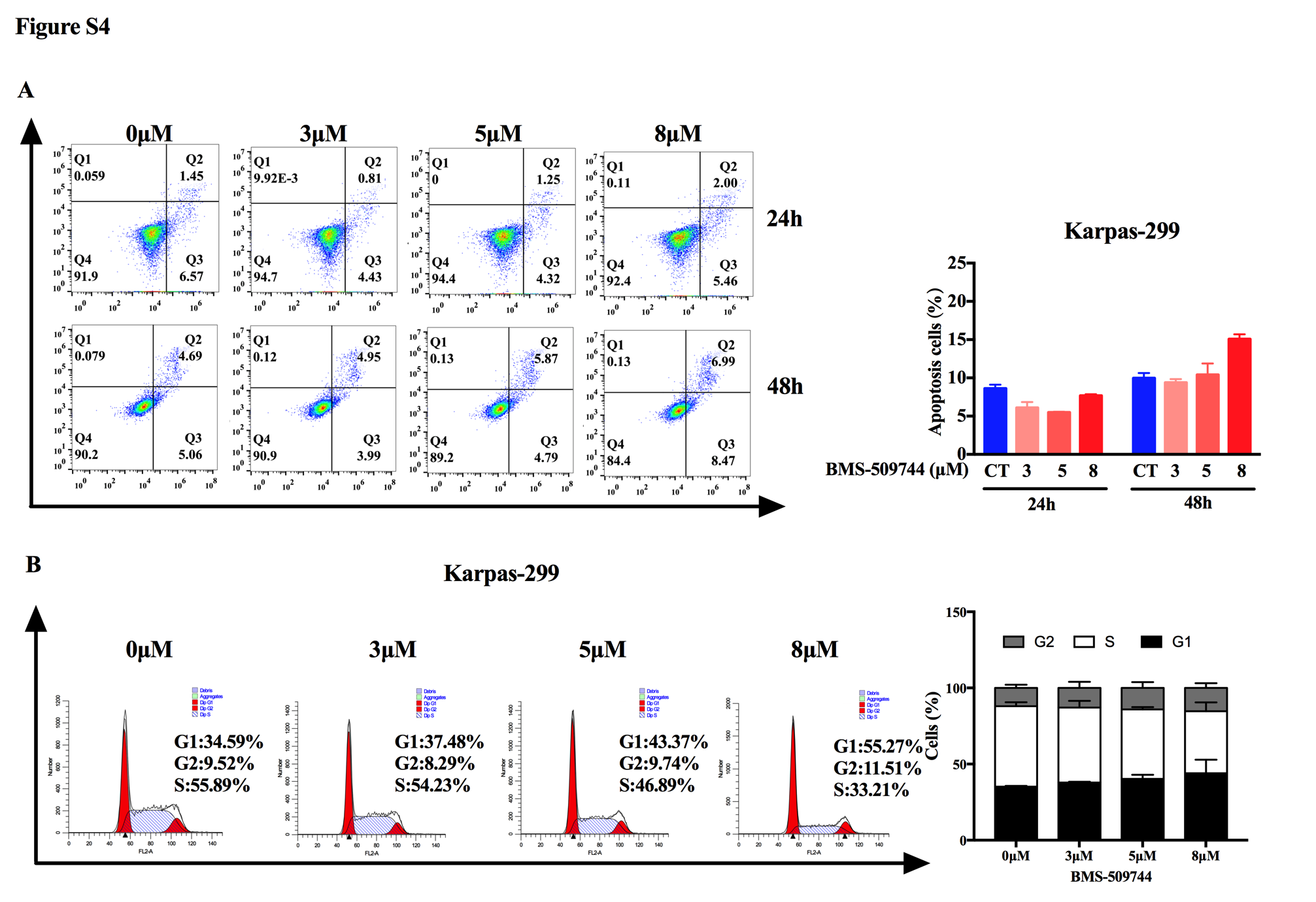

Supplement: Supplementary file 1 — Additional file 1: Figure S1. ITK inhibitor HY-11066 inhibits the proliferation of malignant T-cell lymphoma cell lines. Jurkat, Hut-78 and H9 cells were treated with the indicated concentrations of the ITK inhibitor HY-11066. The cell viability was measured using the Cell Titer-Glo Luminescent Cell Viability Assay. Viable cells (% control) were calculated. Data are expressed as Mean ± SD and representative of three independent experiments. Figure S2. Transwell invasion and migration assays of ITK inhibition in Karpas-299 cells. (A) Karpas-299 cells transfected with vectors carrying ITK shRNA (shITK-34467) or shControl were subjected to invasion assays and migration assays using Transwell chambers coated with Matrigel and Transwell chambers without Matrigel respectively. The cells were monitored by a CellTiter-Glo Luminescent Cell Viability Assay. Data are expressed as Mean ± SD and representative of three independent experiments. Statistical analysis was performed using Student’s t test. *P < 0.05, **P < 0.001 compared with the control group. (B) Migration and invasion assay images of Fig. 3c, d. Figure S3. The chemosensitivity to common chemotherapeutic agents in Karpas-299 cells after the inhibition of ITK. Karpas-299 cells transfected with shITK (shITK-34467) or shControl were exposed to vincristine (A) or doxorubicin (B) for 72 h. Cell viability was measured using a Cell Titer-Glo Luminescent Cell Viability Assay. Data are expressed as Mean ± SD and representative of three independent experiments. Statistical analysis was performed using Student’s t test. *P < 0.05, **P < 0.001 compared with the control group. Figure S4. ITK inhibitor BMS-509744 have no effect on the apoptosis and cell cycle arrest in karpas-299 cells. (A) Karpas-299 cells (2 × 10^5^) were treated with BMS-509744 (3 μM, 5 μM, or 8 μM) for 24 and 48 h, and apoptotic cells were quantified using flow cytometry. (B) Karpas-299 cells (2 × 10^5^) were treated with different concentrations of BMS-509744 ( [file 12935_2019_754_MOESM1_ESM.zip › Figure S4.tiff]
